# A Blueprint for Real-Time Functional Mapping via Human Intracranial Recordings

**DOI:** 10.1371/journal.pone.0001094

**Published:** 2007-10-31

**Authors:** Jean-Philippe Lachaux, Karim Jerbi, Olivier Bertrand, Lorella Minotti, Dominique Hoffmann, Benjamin Schoendorff, Philippe Kahane

**Affiliations:** 1 INSERM, U821, Lyon, F-69500, France; 2 Institut Fédératif des Neurosciences, Lyon, F-69000, France; Université Lyon 1, Lyon, F-69000, France; 3 Laboratoire de Physiologie de la Perception et de l'Action, CNRS, Collège de France, Paris, France; 4 Department of Neurology and INSERM U704, Grenoble Hospital, Grenoble, France; 5 Department of Neurosurgery and INSERM U318, Grenoble Hospital, Grenoble, France; James Cook University, Australia

## Abstract

**Background:**

The surgical treatment of patients with intractable epilepsy is preceded by a pre-surgical evaluation period during which intracranial EEG recordings are performed to identify the epileptogenic network and provide a functional map of eloquent cerebral areas that need to be spared to minimize the risk of post-operative deficits. A growing body of research based on such invasive recordings indicates that cortical oscillations at various frequencies, especially in the gamma range (40 to 150 Hz), can provide efficient markers of task-related neural network activity.

**Principal Findings:**

Here we introduce a novel real-time investigation framework for mapping human brain functions based on online visualization of the spectral power of the ongoing intracranial activity. The results obtained with the first two implanted epilepsy patients who used the proposed online system illustrate its feasibility and utility both for clinical applications, as a complementary tool to electrical stimulation for presurgical mapping purposes, and for basic research, as an exploratory tool used to detect correlations between behavior and oscillatory power modulations. Furthermore, our findings suggest a putative role for high gamma oscillations in higher-order auditory processing involved in speech and music perception.

**Conclusion/Significance:**

The proposed real-time setup is a promising tool for presurgical mapping, the investigation of functional brain dynamics, and possibly for neurofeedback training and brain computer interfaces.

## Introduction

For patients who undergo brain surgery to treat medically intractable epilepsy, the outcome crucially depends on the accurate definition of the brain volume that has to be resected. Becoming seizure-free and maintaining normal neurocognitive functions after surgery are both fundamental in order to improve the patient's quality of life. This requires not only the identification of the underlying epileptogenic network but also a detailed understanding of the functional organisation of the cortical areas in which it is embedded, so that eloquent cortical regions can be spared [1]–[3]. Given the large interindividual variability in functional cortical organisation [4], especially when lesions are present, the precise mapping between the implanted cortical structures and their functions must be performed for each patient individually and may not be solely inferred from previous neurological reports and neuroimaging studies.

The gold standard for assessing the risk of neurological impairment following epilepsy surgery is electrical cortical stimulations (ECS) mapping. This procedure (i.e. [5]) consists in delivering series of short (∼1 ms) low intensity (∼1 mA) current pulses through the intracerebral electrodes to produce localized current flows which interfere with local neural activity. In general, ECS are performed under continuous video-EEG control in several sessions after electrode implantation throughout the pre-surgical evaluation period. Stimulations may reproduce aura symptoms, induce electroclinical seizures, and provide valuable information regarding the organization of the epileptogenic network. Also, they generally reproduce reversibly the consequence of focal cortical lesions, which effect on behaviour, such as speech disruption, can be observed by the experimenter and reveal eloquent areas. Alternatively, stimulations can also trigger positive neural responses, for instance in the sensori-motor cortex where they are used to map the sensori-motor homonculus. The direct effect (be it excitatory or disruptive) of electrical stimulation on the processes carried by the target neural population allows for the mapping of various functional systems such as the sensorimotor system, the vestibular system and neural processes involved in memory and language [1]–[4], [6]–[10].

Furthermore, throughout the presurgical evaluation period, the patient's cerebral activity is continuously recorded via implanted electrodes in order to detect the area of seizure onset. Passive intracranial recordings provide a further attractive setting for functional mapping performed by engaging the patients in various cognitive protocols and analyzing the signals recorded at all the implanted electrodes in search for possible task-related responses [11]. Evidence from a growing body of research investigating event-related dynamics of brain activity in human and non-human primates has revealed that the neural processes underlying motor behavior, perception and higher-order cognitive tasks are associated with neural oscillations in various frequency bands (e.g. [12], [13]). Moreover, low (30–70 Hz) and high (>70 Hz) gamma range cortical oscillations have been shown to display higher spatial selectivity than the lower alpha (∼7–12 Hz) and beta (∼15–30 Hz) cortical rhythms [14]–[16]. The high temporal and spatial selectivity of intracranial gamma power make it a highly robust candidate index for functional mapping. Indeed, cortical networks of gamma band power modulations have been reported in numerous human intracranial recordings, including studies of memory [17]–[19], visual attention and perception [20]–[24], audition [25]–[27], somatosensory and motor processes [14], [28]–[32] and language [33], [34]. Further, a recent study suggests that high gamma (>70 Hz) mapping might be used as a valuable complement to standard ECS mapping during pre-surgical evaluation [35]. That study recorded from thirteen epileptic patients with electrodes distributed over large portions of the left hemisphere and compared gamma band responses induced by the presentation of line drawings in a naming task, with disruptions of naming ability by electro-cortical stimulations. The outcome was that gamma band responses could predict the disruptive effects of ECS with good specificity, leading to the conclusion that gamma mapping could be used to guide ECS during pre-surgical evaluation. Hence, the analysis of intracranial gamma power is of high interest both for presurgical clinical purposes and for basic functional brain research.

Recent increments in computing power and the rising interest in brain computer interfaces (BCI) have fuelled resurgent interest in online analysis of human brain activity including the online estimation of changes in spectral power. For instance, the movement-related mu and beta power modulations (∼10 and ∼20 Hz components) recorded non-invasively with scalp-EEG have been successfully used to implement two-dimensional control of a cursor via motor imagery [36], [37]. More recently, online spectral analysis of electrocorticographic (ECoG) recordings has also been applied within an invasive BCI system and was shown to provide rapid training and stable decoding of movement intentions with, interestingly, a prominent role for high gamma oscillations (up to 180 Hz) in encoding information about the direction of two-dimensional joystick movements [38]. To date, real-time estimation of the brain's spectral power has been predominantly implemented as a component of the procedures used to interface the brain with computers with the primary aim of restoring a degree of mobility to motor-disabled or ‘locked in’ patients [39]–[41] and to investigate neurofeedback-based approaches to hyper-activity, depression, tinnitus [42].

In this paper, we introduce real-time spectral analysis of brain signals to the clinical setting of presurgical evaluation of epilepsy and provide evidence in favour of its use as a complement to standard electrical stimulation mapping and for its potential as an online exploratory tool that can be used to probe spatial, temporal and spectral aspects of cognition. The proposed analysis procedure can be performed either with or without visual feedback to the patient and can lead to novel experimental paradigms that are tailor-designed to systematically test a phenomenon observed during online exploration. As a proof of concept, such an approach is reported here for the case of high-level auditory processing in two epileptic patients. The preliminary findings reveal, for this specific field of brain functions, a highly specific functional and spatial organization of high gamma (60–140 Hz) power in the anterior superior temporal lobe associated with speech and music perception.

## Materials and Methods

### Experimental subjects

The proposed real-time system was tested with two patients suffering from drug-resistant partial epilepsy and candidates for surgery. Because the location of the epileptic focus could not be identified using non-invasive methods, the patients underwent intracerebral EEG recordings using stereotactically implanted depth electrodes (SEEG) (for details see [5]). The selection of electrode implantation sites was solely based on clinical considerations directly related to the presurgical evaluation and without any reference to the present experimental investigations. All patients provided informed consent prior to their participation in the experiment. The detection of task-related brain activations and low-amplitude cortical stimulations were performed by the clinical staff as part of an ensemble of procedures (alongside neuropsychological and psychiatric evaluation) which constitute our standard presurgical clinical routine, not subject to approval by an ethics commitee under French law.

### Intracerebral recordings

A multi-channel video-EEG acquisition and monitoring system (Micromed, Treviso, Italy) was used to simultaneously record the intracerebral activity from up to 128 depth-EEG electrodes (512 Hz sampling rate and a 0.1 to 200 Hz bandpass filter). Twelve to 14 semi-rigid multi-lead electrodes were implanted in each patient. Each electrode had a diameter of 0.8 mm and, depending on the target structure, comprised between 5 and 18 contact leads 2 mm long and 1.5 mm apart (Alcys, Besançon, France). The electrode contacts were identified on the patient's individual stereotactic scheme, and were subsequently anatomically localized using Talairach and Tournoux's proportional atlas [43]. In addition, computer-assisted matching between a post-implantation CT-scan and a pre-implantation 3-D MRI data set allowed for direct visualization of the electrode contacts on the patient's brain anatomy (Voxim, Germany).

### Electrical stimulations

Intracerebral electrical stimulations were performed in several sessions (1 to 2 hours) under continuous video-SEEG monitoring as part of the routine clinical presurgical evaluation procedure. Electrical stimulations are performed to gain more insight into the epileptogenic area but also in order to derive a functional map of the eloquent cortex. Following our standard clinical practice [44], bipolar stimulations at 1 Hz (3 mA) and 50 Hz (1–3 mA) were applied between pairs of neighbouring contacts at different levels along the axis of all the electrodes. The electrical stimuli were delivered using a constant current rectangular pulse generator designed for safe diagnostic stimulation of the human brain (Micromed, Treviso, Italy).

### Real-time visualization of intracerebral power

For the purpose of this study, we implemented a power estimation and visualization software that could be operated in real-time. The program displays the instantaneous variations of signal power at one or several selected intracortical recording sites in one or several predefined frequency bands. The displayed modulation represents an increase or decrease with respect to a baseline level of spectral power which is measured at the beginning of each session while the patient was at rest, relaxed, with eyes open and in a quiet surrounding (∼10 seconds). The spatial specificity of the recordings could be enhanced by switching from single electrode contact data to bipolar recordings computed as the difference between adjacent pairs of contacts.

**Figure 1:**
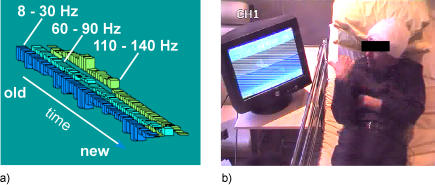
*Brain TV* set-up. (a) The online 3D bar plot displays the time-varying power modulations measured at a selected cortical site over the last 10 seconds in three frequency bands (8–30 Hz, 60–90 Hz and 110–140 Hz). The depicted power modulations represent the power for a given frequency expressed in units of standard deviation of a reference power measured during a baseline period. Baseline power is defined during a calibration phase performed at the beginning of the session while the subject was instructed to rest with eyes open. (Note that the white annotations on the figure are not part of the display). (b) The clinical setting: Patient watching a display that provides him with a visual feedback of online power variations picked up by one of his implanted electrodes.

The real-time analysis and visualization setup consists of the following steps: a) a short-term Fourier transform is applied to the signal (using a sliding Hamming window of 256 milliseconds moving by steps of 128 ms) to extract the time-course of its spectral energy over the last 10 seconds for all frequencies up to 150 Hz, b) the obtained time-frequency (TF) energy map is converted to an Activation score display, that is the energy expressed in units of the standard deviation of the baseline period; in other words energy values at each frequency are normalized with respect to the baseline power level for that given frequency (we remove the mean value of that baseline power, and divide by its standard deviation), c) the normalized TF maps are then averaged in the frequency bands of interest (e.g. [8–30 Hz] and [60–140 Hz], details below), d) the energy time-course is then averaged over a two-second time window to enhance the estimation of the sustained oscillatory components (note, however, that the length of this smoothing window is not fixed. The empirical value chosen here achieved a reasonable compromise between favouring sustained oscillations and preserving a real-time feel to the display). Finally, e) the frequency-specific power variations over the course of the last 10 seconds are displayed on a computer screen. Note that data visualization can be switched to 3D bar plots (figure 1) or to 2D continuous line plots. The implemented software was set to refresh the display as often as possible, that is, to read and analyze newly acquired data as soon as the previous cycle of analysis and display is completed. The real-time program runs under Matlab (The Mathworks, inc) on a PC that can access (or receive) the ongoing recordings made by the EEG acquisition system via an Ethernet connection (or via a TCP/IP protocol respectively). Both power estimation and data visualization routines were optimized to enhance the display refresh rate which is mainly dependent on the rate at which the EEG acquisition system writes the data to the hard drive. For the data reported here, this was done every 250 ms (corresponding to a 4 Hz data update rate).

### Experimental procedures

Various experimental conditions were used to test the feasibility and the possible merits of the proposed real-time system. In principle, the recording sessions using the online display of spectral power modulations can be performed either with or without visual feedback to the patient. During the feedback-based sessions, the patient viewed the spectral power of his/her ongoing intracerebral signals as it varied in time. Such a setting can obviously be used to set up neurofeedback training sessions during which the subject attempts to use the visual feedback to control certain aspects of his/her cerebral activity or mental state. This was however not attempted in the current study. Instead, we tested the neurofeedback approach as a putative exploratory tool allowing the patient to search for correlations between the time-varying display of his/her brain activity and endogenous as well as exogenous events. These exploration sessions consisted of two parts: a) a ‘free search’ session during which the patient is left alone with the functioning display so as to explore at his/her own leisure, with no a priori information, possible relationships between the display and his/her behavior and b) a ‘guided search’ session, during which the experimenter encourages the patient to test or pay attention to broad cognitive processes that are likely to be carried by the cortical area under investigation (e.g. sound perception for the auditory cortex). The instructions of the ‘guided search’ session are based on the existing functional imaging literature and, if available, on results of previous cognitive experiments the patients may have participated in.

This feedback-based procedure largely relies on the patient's motivation and his/her ability to detect and accurately report correlations. This makes this approach both complex and adds a subjective dimension to the reported phenomena. Therefore, the real-time sessions were complemented by experimental paradigms designed to confirm and provide a statistical analysis of the functional correlations reported by the patients during the online sessions. These experiments were custom-designed to specifically investigate the correlations considered to be most robust (according to the patient and/or experimenter) and were analyzed off-line using spectral analysis techniques and tested for statistical significance.

In summary, the experimental procedures used in this study consist, on the one hand, of real-time functional exploration sessions either with or without visual feedback to the subject and, on the other hand, of controlled follow-up experiments custom-designed to systematically test prominent reports of correlation between the ongoing power variations and the patient's behavior. Given that the design of these *follow-up experiments* was entirely prompted by the nature of the real-time observations, they represent a direct result of the online findings and are therefore, for the sake of consistency/readability, described after the results of the real-time investigation framework.

### Data analysis of follow-up experiments

Since the aim of the follow-up protocols was to verify the observations made (by the patient and/or by the experimenter) during the online experiments, the offline analysis was set to be as close as possible to that used during the real-time power estimation sessions. That data from each trial was analyzed in the time-frequency (TF) domain investigating frequencies up to 200 Hz across a time window starting 3 s before and ending 2 s after a given event marker (generally occurring at the end of a continuous stimulus period). Note that the frequency range extended higher than during online sessions (200 Hz vs. 150 Hz), because no limitation of computing speed needed to be taken into account in this offline analysis. The single-trial TF energy maps were normalized independently for each frequency with respect to the mean and standard deviation of a common baseline (0 to 200 ms). Note that although the TF maps were averaged across all trials in a given experimental condition for visualization purposes, the statistical analysis was performed on the single-trial TF maps. The specific bounds of the frequency bands of interest (e.g. alpha/beta, gamma etc.) and the width of the averaging time-window (e.g. 2 s), which had to be defined prior to the statistical testing, were selected in accordance with the values that had been reported to yield the strongest effects in the real-time analysis sessions.

### Statistical analysis

The statistical significance of the time-frequency energy values computed from the data collected during the follow-up experimental paradigms was performed with a nonparametric Kruskal-Wallis (KW) test and Fisher's protected least significant differences based on ranks (FPLSD) for post hoc tests of significance (Portney and Watkins, 1993). The Kruskal-Wallis tests were performed on ranked data and provided a H-value, i.e. an indicator of whether a global effect of a given stimulus type is significant. If an effect was found to be significant with the KW method, FPLSD tests were then used to compare all the possible combinations of experimental condition pairs, and thus determine the pairs for which significant differences occurred.

## Results

In the following, we first report the findings of the real-time sessions and then describe the design and results of the follow-up offline experiments that they elicited.

### Real-time intracerebral power analysis

Both patients were able to use the real-time power analysis setup to follow the ongoing modulations of the spectral power of the recordings of a chosen depth electrode contact in two distinct frequency bands simultaneously. The two displayed frequency bands were selected by the experimenter at the beginning of each session, however, the choice of the electrode for which the spectral power was displayed on the screen was made by the patients themselves during the ‘free search’ sessions and by the experimenter during the ‘guided search’ sessions. The patients used a remote control device to switch the display through various available SEEG channels depicting the ongoing spectral power measured at various locations within their brain (which led us to call the system “Brain TV”).

The neurofeedback consisted of presenting the patients with a display of their brain activity (figure 1). The display appeared as a 3-D dynamic bar plot depicting the relative modulations (over the last ten seconds) of the estimated power in the alpha/beta range (8–30 Hz) and in the high gamma band (60–90 Hz and 110–140 Hz, thus avoiding contamination by the mains 50 Hz frequency and its harmonics). The alpha and beta bands were pooled into one interval because initial real-time plots using distinct alpha and beta representations showed strong covariations of power in these frequencies for most of the electrodes investigated here, inline with previous reports investigating task-related neural oscillations during both sensory and motor tasks [15], [21], [33]. By contrast, the high gamma band modulations appeared to display a behavior largely contrasting with that of the lower bands. Moreover, we decided to restrict the number of visualized frequency bands to 2 or 3 bands at a time to keep the displayed visual information as simple as possible. Most importantly, at the beginning of the session, the real-time power estimation system was first of all calibrated by automatically estimating a baseline power value in each frequency band, based on data acquired during a 10 seconds resting period.

### Real-time high gamma power modulations

The online feedback-based sessions started with a ‘free search’ period, during which we simply let the patient get used to remotely operating the online display (channel selection) and become familiar with its variations over time. The patient was informed that the display represented the activity of a given region of his/her brain in real time, but no information was given with respect to the location of the visualized electrodes or the putative function of the various implanted cortical areas. We encouraged the patients to pay attention to possible temporal correlations between changes in the display and events in the environment or in their own experience, both at rest and during usual activities such as, for example, moving, speaking or reading. Given that the display represented the temporal evolution over the last 10 seconds of cortical activity, the patients easily learned to switch attention from their behavior to the display in order to detect possible correlations. Furthermore, the software also allowed the experimenter to add a large time lag of 30 s to the display, without informing the patient, in order to test the reliability of a patient's reports of correlations between his/her behavior and the viewed power changes.

#### Patient One

For patient 1, the ‘free search’ period lasted 10 to 15 minutes during which the patient became familiar with the system and choose to look out for the effects of relaxing and reading on the real-time power plots. This was prompted by the fact that the patient had previously participated in a cognitive paradigm involving silent reading of words and pseudo-words. At the end of the ‘free search’ period, conducted in silence, the experimenter entered the room and asked the patient if he had noticed any systematic relation between his behavior and his visualized brain activity. The patient reported that he was unable to identify any clear correlations either for relaxation or for silent reading irrespective of the electrodes he selected. However, the conversation itself triggered a strong increase of the online gamma power index. The repetitive increase of high gamma power during the ongoing conversation between the patient and subject was evident at the electrode contact t'8 and was first interpreted as a general arousal effect but quickly turned out to be more specific. Several minutes of exchange between the experimenter and the patient followed. In order to address a possible relationship between the online power indices and reading or speech perception, the patient and the experimenter took it in turns to read aloud. After this period, it seemed increasingly clear that the high gamma power index was reacting to the experimenter's speech, as if high gamma oscillations at the location of t'8 were functionally involved in the perception or processing of speech produced by another person. Note that the electrode site t'8 was located in the mid part of the upper bank of the left superior temporal sulcus (mSTS) (Talairach coordinates : −65, −12, 4 mm).

The ‘guided search’ session went on for another 30 minutes, during which multiple speech-related tests were performed by the experimenter (and several members of the clinical staff). These tests included repeating nonsense speech patterns (e.g. ‘patapatapata’), or talking to the patient in multiple languages. While, the real-time gamma power index appeared to remain unaffected by hearing nonsensical phrases, it invariably increased while the patient listened to a person speak. Interestingly, the gamma power elicited by speech revealed a tendency to be differentially modulated by the patient's knowledge of the language. For instance, the gamma response was stronger for German, which the patient understood at school-level, than for French. In contrast, no comparable gamma modulations were observed while the patient spoke out loud nor while he listened to loud noise (such as drumming patterns). In addition, no real-time gamma modulations were visible during perception of predictable speech patterns such as the alphabet. At the end of this session, we came to hypothesize that, at the location of the mSTS region probed by electrode t'8, high gamma power increases were functionally related to the phonological processing of spoken sentences. A tempting hypothesis that we further investigated with a follow-up paradigm and statistical analysis.

### Follow-up auditory paradigm

To investigate in more detail the functional nature of the gamma power modulations in mSTS (electrode t'8) suggested by the real-time session described above, we designed a paradigm that consisted of six experimental conditions: Four auditory processing conditions during which the patient listened to sentences pronounced in his native language (1.MOTH_EXP), in a language unknown to him, (we used Finnish) (2.SUOM_EXP), to repetitive nonsensical speech patterns such as ‘patapatapata…’ or ‘mumomumomumo….’ (3.PATA_EXP) or loud noises (we used percussive drum patterns) (4.NOIS_EXP). Additionally, the paradigm included two speech production tasks in which the patient was instructed either to read sentences aloud (5.READ_PAT) or talk freely, for example pronouncing improvised sentences describing recently past events (6.TALK_PAT) both in his native language. These conditions were designed to mimic the tests performed in real-time and thereby provide a statistical evaluation of the functional phenomenon detected via our real-time exploration. The protocol was block-designed, with 10 to 20 stimulus periods of about 10 seconds for each condition. Stimulus periods were separated by at least one second and a time-marker indicated the end of each stimulus period and was used for averaging.

**Figure 2:**
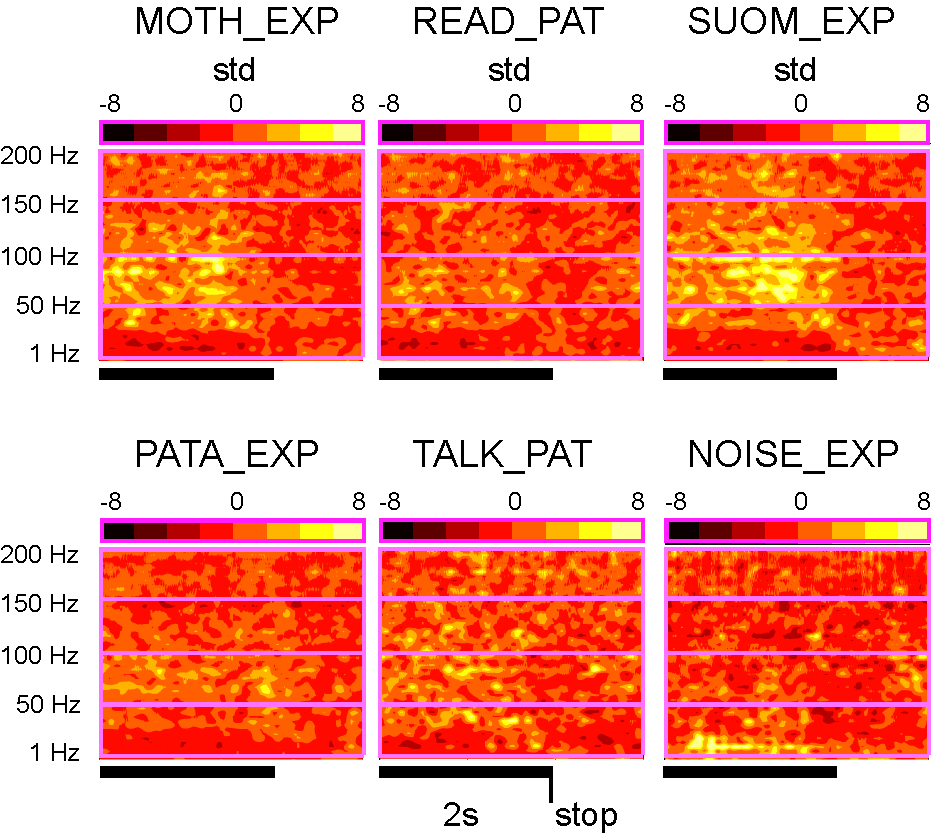
Oscillatory power modulations associated with speech in the mid superior temporal sulcus. Time-Frequency maps representing the time course of spectral power modulations at electrode t'8 (Talairach coordinates : −65, −12, 4) for frequencies up to 200 Hz in each one of the 6 experimental conditions. The frequency values range from 1 to 200 Hz and the horizontal time axis spans over 3 seconds including 2 s of auditory stimulation (black horizontal bar). Note that, power modulations are given in units of the standard deviation from baseline period (see methods) and that the normalization parameters were the same for all maps.

Figure 2 shows the time-frequency energy maps for these six conditions, normalized relative to a common baseline. The maps, which represent the energy averaged over the multiple trials of each condition, show a decrease in gamma band activity after the end of the sentence predominantly in the first three conditions, but not in the last three. In other words, gamma oscillatory power is enhanced during the conditions involving the perception of speech, but clearly less so during perception of noise or during speech production.

**Figure 3:**
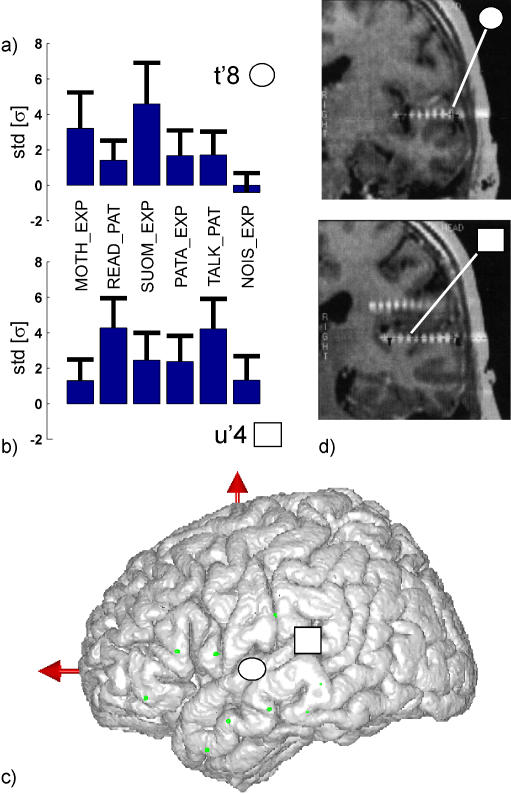
Differences in gamma band reactivity between electrodes in mid superior temporal sulcus and the primary auditory cortex. (a,b) Bar plots of mean and standard deviation of the oscillatory power increase measured in the gamma range (60–140 Hz) during the last two seconds of auditory stimulation, for all six experimental conditions at two distinct locations: The upper plot (a) represent the modulations obtained from electrode t'8 in the mid superior temporal sulcus (Talairach coordinates : −65, −12, 4), while the lower plot (b) corresponds to site u'4 in the primary auditory cortex (Talairach coordinates : −43, −27, 11) . (c) Position of the two electrodes (t'8 and u'4) when displayed on a standard 3D cortical rendering of the Montreal Neurological Institute (MNI) single subject and (d) as seen on the patient's individual MRI.

To quantify this effect, we examined the task-related high gamma power changes recorded at site t'8 by pooling the power estimations for the [60–90 Hz] and [110–140 Hz] ranges (avoiding the 100 Hz power supply artefacts) and averaging over the last two seconds of each epoch (as was done with the indices visualized in the real-time analysis). The gamma band power modulations (relative to baseline) elicited during each of the six conditions are displayed in figure 3 as bar plots of mean and standard deviation (std). The two highest gamma responses were obtained with the foreign and native language perception tasks with the former showing the strongest deviation from baseline. By contrast, the noise perception task was associated with a task-related decrease in relative gamma power.

Statistical analysis revealed a significant global effect of the experimental condition (Kruskal-Wallis, H = 45.7, p<0.0001) and the high gamma power was found to be significantly higher in the foreign language auditory processing condition (SUOM_EXP) than in all other conditions (p<0.05, Post-hoc FPLSD test) except the native language auditory perception task (MOTH_EXP). Using the same test, the gamma power in the latter condition was also found to be significantly larger (p<0.05) than in the noise perception condition (NOISE_EXP). Moreover, the difference between native and foreign language processing tested significant (SUOMI_EXP>MOTH_EXP, p<0.05) with a Mann-Whitney comparison.

The above analysis was focused on the data from electrode t'8 as this was the electrode for which the effect was found in the real-time analysis session. For the sake of comparison, we also examined the same measure of gamma power modulations for a recording site located in the left primary auditory cortex (Heschl's gyrus) (electrode u'4, Talairach coordinates : −43 ,−27, 11 mm, see Figure 3), an area known to display activations related to lower level processing of auditory stimuli. Although, the mean power bar plot (figure 3) reveals that there are also significant task related gamma power modulations between conditions at this electrode (Kruskal-wallis, H = 37.7, p<0.0001), the responses were different from that observed in the mSTS. Indeed, Post-hoc FPLSD testing showed that the strongest energy was obtained in the two conditions in which the subject talked (TALK_PAT>MOTH_EXP and NOIS_EXP, p<0.05) and read out loud (READ_PAT>SUOM_EXP, PATA_EXP, MOTH_EXP and NOIS_EXP, p<0.05).

### Electrical Cortical Stimulations versus real-time gamma modulations

Given the relevance of the above findings to the functional mapping aspect of the presurgical evaluation period, we compared the real-time gamma power results with the report of the electrical cortical stimulation (ECS) sessions that was carried out with this patient beforehand. We found that the initial ECS session had revealed no particular effects when stimulating the site t'8. During stimulations at this location (mSTS), the patient had been asked to count aloud, or to listen to a member of the medical staff count, and then he was asked whether he experienced any problems in correctly hearing the counting. The patient reported no particular difficulty which suggested no auditorily disruptive effect caused by stimulating this electrode either at 1 Hz or at 50 Hz (with amplitudes up to 3 mA). However, the results of the real-time power analysis sessions (later confirmed by the offline analysis) indicated that this area was likely to be involved in speech processing. In fact, it showed an activity specific to hearing non-predictable speech patterns, unlike counting. This led us to stimulate this site in a second ECS session during which we specifically tested the patient for speech perception impairment by means of actual sentences. Indeed, this time, we noticed that 50 Hz stimulations (3 mA) produced difficulties in sentence comprehension. For comparison, stimulations of u'4 at 1 Hz (3 mA) had no such disruptive effects on sentence understanding, but stimulations of this region at 50 Hz (1 mA) triggered auditory illusions, described by the patient as ‘echo’ whereas applying the same stimulation to a site 15 mm more medial to u'4 triggered auditory hallucinations, i.e. perception of high-pitch sounds. On the whole, the additional ECS session that was triggered by the real-time gamma power findings in this patient helped refine the functional mapping and might be seen as an example of how the two techniques can be complementary.

The patient was operated on eight months ago, and he has remained seizure free since then. Surgery consisted in a left anterior and mesial temporal lobectomy that spared the mid and posterior part of the first temporal convolution, in which electrodes t'8 and u'4 were located, respectively. There was no post-operative deficit, including verbal expression and comprehension.

#### Patient Two

The usefulness of the real-time system was further investigated with a second patient who had 14 implanted electrodes. In contrast to patient P1, patient P2 had electrodes implanted in the right as well as in the left perisylvian regions. This experiment largely focused on the anterior superior portion of the temporal lobe and followed a procedure identical to the one described for patient P1 with, in this case, two follow-up experimental protocols adapted to the results of the real-time session.

In the guided search session we investigated the reactivity of the electrodes recorded from the mid portion of the superior temporal gyrus (bilaterally) initially testing for reactions to speech similar to the ones observed in P1. Such effects were not apparent via the real-time analysis in this patient. Although this is most probably due to differences in anatomical location, it could also be explained by inter-individual variability in cortical organization of higher-order auditory processes or possibly by the fact that the amplitude of the effect was not strong enough for it to be detectable using online single-trial analysis. Compared to the t'8 electrode in P1, the mSTS electrodes examined for speech effects in P2 were slightly more anterior and superior and did not seem to reach the STS proper. However, further exploration (alternating between guided and free search) rapidly (15 to 20 minute session) yielded two distinct and intriguingly strong correlation effects. First of all, we found that music and speech triggered sustained high gamma band responses in spatially distinct cortical sites (interestingly, this was found in both hemispheres : sites t'6 and t7, respectively in the left and right mSTG, generated a stronger response to speech, while sites t'4 and t5, which were located 7 mm more medial than t'6 and t7 respectively, in the left and right mSTG, generated a stronger response to music). The second robust observation was that the gamma band activity recorded at site u'8 in this patient, in the posterior portion of the left STG (Wernicke's area), in a region classically associated with social cognition, seemed to increase in power in response to changes in speaker. The power increase in this site seemed selectively triggered by speech onset and vanished after less than two seconds as the speech continued. Furthermore, we observed that this transient response to speech onset was less obvious when the same experimenter would resume speaking after a short pause. In contrast, the response was much stronger when a new speaker (another experimenter) would next talk. To test for this effect ‘online’, three experimenters read in turn the same sentence, through four such speech cycles and found that the beginning of each sentence was systematically associated with the generation of a strong transient response in u'8. In contrast, the response was reduced to zero when the same experimenter read the same sentence several times.

### Follow-up experiments

To test for these speech and music related high gamma responses in a controlled manner, we designed two experimental paradigms. In the first protocol we set out to test for the speech versus music selectivity. The paradigm consisted of the following 8 conditions: Condition “SPEECH”: a passive listening task, during which the patient listened to 7 seconds-long sound sequences containing 10 sentences pronounced by computerized female and male voices. Condition “FLIPPED”: the same 10 sentences reversed in time. Condition “SUOMI” : 10 sentences in a language not spoken by the patient (we chose Finnish) pronounced by the same computerized voices. Condition “COUNT”: 10 predictable number lists (countdowns and countups). Condition “PATA”: 10 repeated nonsensical speech patterns (‘patapata’). Condition “MUSIC”: 10 instrumental music excerpts. Condition “OPERA”: 10 excerpts of Opera singing. Finally, condition “SUBJECT”: 10 additional computer voiced sentences were presented to the patient during which she was asked to count aloud in order to mask the sound of the computer voice. The sound sequence presentation order was randomized with a 3 s interval between consecutive sequences and sound sequences were delivered binaurally via earphones via the *Presentation* stimulus delivery software (Neurobehavioral systems Inc). All acoustic signals had the same intensity, quantified by their root mean square, set at confortable sound level.

**Figure 4:**
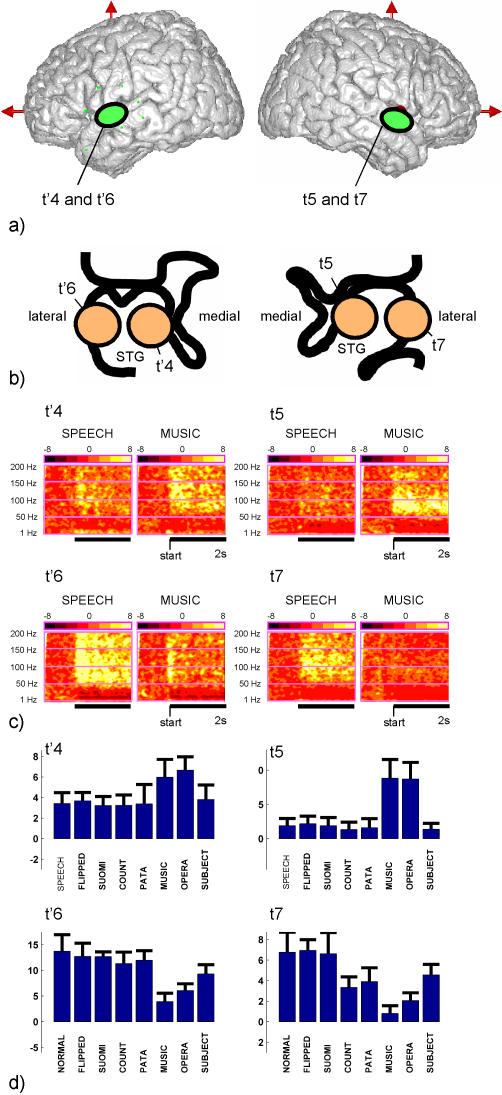
Functional dissociation between regions of the mid portion of the superior temporal gyrus in response to speech and music. (a) The general region in which each site was located is represented with green oval markers. Note that this patient had a cardiac pacemaker incompatible with MRI scanning and therefore, the Talairach coordinates of the sites could not be reconstructed with a precision higher than 1 cm along the y and z axis. (b) The spatial location of the recording sites in the coronal plane relative to the Sylvian fissure. (c) Time-Frequency maps depicting the time course of spectral power measured in left (t'4, t'6) and right (t5 and t7) sites for all frequencies up to 200 Hz during the first two seconds of auditory stimulation (black horizontal bar) in response to speech (SPEECH) and music (MUSIC). The baseline is common to the NORMAL and MUSIC conditions. (d) Bar plot display of the mean and standard deviation of the total activation measured in the high gamma (60–140 Hz) range during the first two seconds of auditory stimulation, for each one of the eight conditions of the SOUND paradigm.

The oscillatory responses to the various sounds sequences of this experiment for 4 sites in the left and right mSTG (electrode sites: t'4, t'6, t5 and t7) are shown in figure 4. Our results show that the gamma band response (60 and 140 Hz) measured during the first two seconds of stimulation was stronger for normal speech than for music in the more medial sites bilaterally (i.e. at sites t'4 in left mSTG and t5 in right mSTG, Mann-Whitney U test, p<0.01), while it was stronger for music than normal speech in the two more lateral sites (t'6 in left mSTG and t7 in right mSTG, Mann-Whitney U test, p<0.01). These findings are visible in the gamma response bar plots (figure 4 d) as well as in the Time-Frequency maps (figure 4 c). The latter also show that the high gamma band response recorded at those sites was sustained in time.

The second protocol (SPEAKER) was designed to test for the effect of speaker change on the cortical activity measured via electrode u'8. It consisted of a passive listening task, during which sound sequences were presented in pairs (two 7-s long sound sequences separated by 3 seconds). The study examined the response to the second sound of a pair, depending on the nature of the first one. There were six types of pairs : in the SN1 pairs, the first and second sounds were two different sentences spoken by the same speaker (the condition code refers to what changes between the two sounds, SN = sentence, SP = Speaker, MUS = Music); in the SN pairs, the first sequence was a time-reversed sentence and the second sequence the same sentence (not reversed) spoken by the same speaker; in the SN+SP pairs, the first and second sequences were two different sentences spoken by different speakers; in the SP pairs, the first and second sequences were the same sentence spoken by different speakers; in the MUS1 pairs, the first sequence was a normal sentence and the second sequence a music excerpt; in the MUS2 pairs, the first and second sequences were two music excerpts played by two different instruments. Between two consecutive pairs, a 5-s long musical jingle was delivered to limit the memory effect of the last sound sequence of a pair onto the first sequence of the next pair. All sound sequences were preceded by a 3-s silence. Twelve sound pairs of each kind were presented, and the order in which they were delivered was random. In both experiments, all sound sequences were delivered binaurally via earphones with the *Presentation* stimulus delivery software (Neurobehavioral Systems Inc) and all had the same RMS (root mean square) at comfortable sound level.

**Figure 5:**
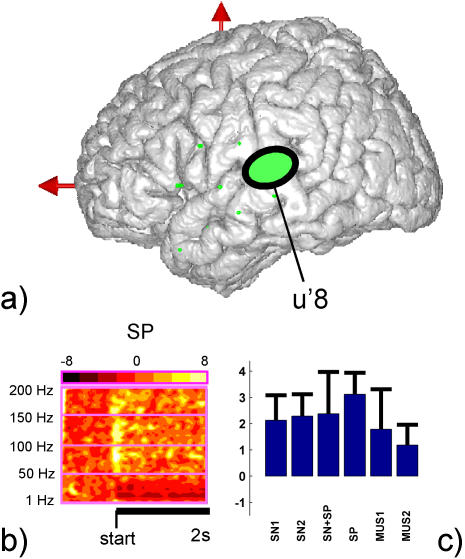
Response to “speaker change” in the posterior part of the left superior temporal gyrus. (a) Location of the electrode site u'8 (green oval marker) (b) Time-frequency map depicting gamma response to a sentence uttered by a new speaker (second sentence in a SP pair). (c) Bar plots illustrating gamma response during the first two seconds of auditory stimulation for the second sound sequence of each type of sound sequence pairs in this protocol. (see paradigm description in the text for more details)

The results of the second experiment designed to test the response properties of site u'8 are shown in figure 5 and show the effects observed in this area (posterior portion of the STG) for each type of sound transition. The gamma band response was indeed very transient (back to zero after 500 ms) and strongest in the conditions which involved a change in speaker, in particular when the same sentence was pronounced by two different speakers (SP>SN1 and SP>SN2, Mann-Whitney U test, p<0.05; see figure 5). The weakest response was associated with the conditions of changes involving music, showing that the response was probably not simply triggered by novelty. These significant response properties suggest that high gamma oscillations in this area (posterior STG) might play a functional role in the neural processes involved in the detection of a novel voice or of a switch in speaker. This finding will have to be further investigated in more patients with similar implantations or in healthy populations using non-invasive techniques. It is important however to note that this very specific phenomenon would not have been suspected had it not been for the real-time exploration procedure. On the whole, similarly to the experiments in patient 1, the follow-up paradigms in patient 2 yielded a statistical confirmation of the effects detected with the real-time exploration.

#### Electrical Cortical Stimulations

The ECS session in this patient was complicated by the fact that the cortical tissue was very sensitive to electrical stimulations, producing post-discharges even at relatively low current amplitudes. Still 50 Hz stimulations in sites discussed above produced conscious manifestations (1 Hz stimulations produces no noticeable effects) : in t5 (1 mA), the patient could no longer understand the text that the experimenter was reading; also, while music was played, she felt a ‘weird sensation of echo’, as if the music came from two different sources with a delay. However, those results should be qualified by the fact that those stimulations generated a post-discharge. In t7 (3 mA), she felt that the voice of the experimenter changed, as if coming from under a blanket; in addition, she had the feeling that her own voice changed in pitch; stimulations also produced a post-discharge. There were no noticeable effects when stimulating t'4 (3 mA), even while the patient was listening to music. In t'6 (3 mA), the impression was that the male voice of the experimenter changed to sound more like a female voice; and stimulations triggered a post-discharge. Finally, stimulations of u'8 (2 mA) produced the feeling that her own voice drifted away from her (with a post-discharge). In sum, ECS indicated a possible involvement of those brain regions in the perception of music and speech, but the excitability of the tissues prevented more precise functional mapping.

The patient was operated on (left anterior and mesial temporal lobectomy that spared the electrodes studied) and she experienced transient words-finding difficulties in the immediate post-operative phase. Seven months after surgery, there was no more deficit and she was seizure free.

## Discussion

The current report investigated the feasibility and utility of real-time visualization of real-time task-related modulations of oscillatory activity within the clinical setting of intra-cerebral recordings from implanted patients suffering from intractable epilepsy. The results obtained with two patients, presented here as illustrative cases, led to the investigation of higher-order auditory processing related to speech perception. Although the findings obtained with only two patients can be considered to be preliminary, they show that the proposed real-time power analysis approach may lead to the investigation of neuronal processes which would not have necessarily been probed otherwise. Besides, our results suggest that real-time analysis of oscillatory brain activity from intra-cerebral data leads to additional functional information complementing, not replacing, the results of the routine clinical pre-surgical mapping procedures. As such, this study represents proof of concept for the implementation and utility of real-time visualization of task-related high gamma power variations.

So far, the online analysis of task-related spectral power (which is the basis of several BCI applications) has been predominantly restricted to frequencies up to 50 or 60 Hz and/or to well-defined motor or sensory areas [38], [45]–[47]. The fact, that high gamma (from 60 up to 200 Hz) analyses of ongoing brain activity have only been reported in a limited number of studies is mainly due to the fact that these are not easily discernable in scalp-EEG recordings. This is assumed to be at least in part due to the fact that higher frequencies are likely to be generated by smaller cortical assemblies than lower frequencies [14], [48]. In the first study to report the application of electrocorticography spectral power to online operation of a BCI system, Leuthardt et al [38] used online estimations of sensorimotor rhythms up to ∼60 Hz from subdural grid recordings to achieve highly accurate one-dimensional cursor control. Furthermore, during offline analysis using a neural network, the authors showed that two-dimensional movements were best reflected in the high gamma range (up to 180 Hz). In comparison, the gamma band power analysis reported in our study was examined in real-time and, more importantly, was not used in a BCI framework but rather as an exploratory online functional mapping tool.

The cortical origin of the gamma band modulations reported here is largely consistent with the known implication of the mid superior temporal sulcus in speech perception. Indeed, several neuroimaging studies have established that this region is activated in situations contrasting vocal vs. non vocal sounds [49], [50], or human vs. animal vocal sounds [51]. In an fMRI study comparing short vocal vs. non-vocal sounds [49], the maximum BOLD difference was found at a location only a few millimetres away from our t'8 recording site in patient 1 (Talairach coordinates: −62, −14, 0). However, the precise functional role of this region in speech perception is still a matter of ongoing debate [52]. Several studies have proposed that it might be involved in sentence processing [53], [54] either for the syntactic or for the semantic analysis of sentences [54]. In the patient we examined (patient 1), this region responded strongly and selectively to sentences pronounced by the experimenter (both in languages the understood and in a language he didn't), but less strongly when the patient spoke or read a sentence. Non-sentence speech patterns (such as the alphabet) did not generate a comparable response at this location either. This response pattern is consistent with a participation in high-level phonological decoding, which would explain why there was no response to the subject's own voice or to perceiving noise or non-sentence auditory stimuli. However, it is unlikely that the region participated in semantic processing because the strongest response was obtained for sentences in a language unknown to the patient (Finnish). Interestingly, the activity in this region has been found to be altered in autistic children [55]. This might explain certain difficulties in communicating with others. Our results, contribute to the ongoing debate of the role of mSTS in speech processing by providing data from intra-cranial recordings in Humans and suggesting a prominent role for high gamma oscillations in the involved processing.

It is important to keep in mind that the high-gamma findings related to higher-auditory processing presented in this study are limited to single subjects. This is a limitation strongly inherent to the particular setting of clinical intra-cranial data acquisition. Access to human intracranial recordings is limited and, more importantly, the location of the implanted electrodes varies substantially among patients depending on the nature of the epilepsy and on the suspected epileptogenic foci. This makes group level analysis with implanted patients quite difficult. Nevertheless, we hope to further validate our findings by carrying out identical auditory experiments with future patients that would happen to have comparable implantation sites. Additionally, the aforementioned limitation is not critical in the context of evaluating the clinical utility of real-time spectral analysis of intracerebral EEG in individual patients.

A further issue, which is not limited to this report but rather inherent to all previous studies on human intracranial data, is the possible interference between pathological effects and normal neural phenomena. To what extent can we be confident that the reported results do not at least partially reflect pathological effects? Such an effect cannot be totally ruled out given that the location of the electrodes was selected in order to detect and monitor the suspected epileptogenic foci. If the aim is to map eloquent cortex in a given patient, this is not problematic. However, if the aim is to infer about the normal functional organization of the cortex, what can be done, a posteriori, is to verify whether the electrode positions for which we have detected significant task-related modulations were diagnosed as being within the direct vicinity of seizure foci. This verification can be done once we have the results of the 1 to 2 week presurgical evaluation period as well as the post-operative clinical report indicating which brain tissue was indeed pathological. The implantation of depth electrodes is restricted to pharmacologically intractable epilepsy and the aim of the implantation is precisely to detect the epileptogenic foci. In other words, several-sometimes widely separated-structures are implanted for monitoring purposes. These include the epileptogenic foci but also healthy tissue. Differentiating between the two is the main goal of the presurgical evaluation [56]. In the present study, the clinical evaluation and post-operative report confirmed that the areas for which we reported task-specific phenomena were not part of the epileptogenic network.

Although this study reports novel insights into the putative functional role of high gamma oscillations in specific higher-order auditory processing, the primary aim of this paper is to introduce a real-time spectral estimation system and show its potential utility both from a clinical and a research perspective.

From a clinical point of view, the ability to passively probe the implanted areas for a possible involvement in eloquent behavior can be of major utility in the context of presurgical evaluation of epilepsy. In the clinical setting, the proposed system may be used as a complement to the routine tests performed by medical staff in the pre-surgical mapping phase. In particular, ECS results, clearly the gold standard for presurgical mapping, might be complemented and extended with findings from real-time power analysis. This complementarity was particularly evident in patient 2, for whom ECS triggered post-discharges in STS cortical tissue even at low current amplitudes, which prevented a fine functional mapping with this technique. In patient 1, real-time based findings triggered further ECS testing and fine-tuned the presurgical mapping : the observations made during real-time gamma power visualization in this patient prompted the clinical staff to perform additional electrical stimulations to specifically test the cortical area probed by electrode t'8 for its involvement in speech processing. The additional ECS session revealed a disruption of normal speech comprehension during stimulation, an effect that might have otherwise remained undetected. Thus, real-time gamma power analysis may yield observations that provide further functional mapping hypotheses to be tested with ECS. While routine ECS tests rely on a standard functional mapping body of knowledge based on decades of literature, additional ECS sessions triggered by findings from passive real-time power measurements could test for cognitive processes specific to the patient and to the exact position of his/her implanted electrodes. Therefore, the findings reported here provide examples of how online functional gamma power visualization and ECS can be used together interactively to improve the accuracy of the overall presurgical mapping.

### Role of visual feedback

From a basic research perspective, the ability to assess in real-time the task-related power changes of a wide range of brain rhythms at multiple cortical locations is of major interest and can be extremely informative whether applied with or without visual feedback to the implanted patient. Application without feedback allows for the investigation of the functional role of the cerebral tissue in the vicinity of each electrode contact by monitoring power variations online as the patient performs specific instructed tasks or as he/she responds to various stimuli. Spectral changes that can be replicated by repeating the same instruction or by presenting the same stimulus to the patient may indicate a functional role for the observed oscillations and may thereby serve as basis for the immediate design of a controlled experimental paradigm to test the statistical significance of the observed effect. Using the online setup without visual feedback to the patient can be mandatory in certain experimental conditions such as those involving the presentation of visual stimuli. Moreover, it is important to keep in mind that, although feedback-based real-time investigation of human intracranial data might be clinically and scientifically very attractive, some patients might find such an experience overly disturbing and prefer not to have access to graphic representations of their ongoing brain activations.

By contrast, using our real-time system to provide patients with visual feedback of their ongoing cortical power variations opens up possibilities for a range of applications. First, the patient can be left to explore the oscillatory activity of his/her brain in search of causal relationships between the real-time display and a large range of possible parameters including various mental states and simple behavioral conditions that are not necessarily part of the tests generally performed by either researchers or clinical staff. However, although one might expect this approach to be promising for the auto-investigation of a range of sensory and motor processes, it is not yet clear whether it is adapted for the study of more endogenous mental events involving, for instance, emotional or attentional modulations.

To a certain extent, the limits of the feedback-based self-exploratory approach proposed here are largely dependent on two factors: first of all, the introspective capacity of the patient to identify in his/her own subjective experience events that are correlated in time with the displayed neural index and, secondly, the intrinsic variability of the neural signals.

The first issue is at the center of a body of research known as ‘neurophenomenology’ [57]–[60] which explores the advantages and limits of combining ‘first-person data’ (subjective reports) with ‘third-person’ neuroimaging data. Such an undertaking faces several methodological challenges [59] and the assistance of an expert in guiding the patient's introspection can be very beneficial [61]. Another point raised by the neurophenomenological approach is the extent to which the experimenter may trust the subject's reports [62], an issue which becomes even more serious when the online neural index correlates with mental events accessible only to the patient. As is the case sometimes with ECS functional mapping, the experimenter has little option but to take the patient's report as truly reflecting his/her experience. However, it can still be tested objectively whether the patient actually detected some type of correlation between his/her behavior and the observed display. This can be done by artificially adding an important delay to the displayed data (catch trials) and see whether the patient notices it-or simply by removing the visual feedback (turning the screen away from the patient) and checking whether he's able to describe what occurs on the screen when he/she voluntarily generates the given behavior. Nonetheless, all the uncertainties related to the limitations of the online search (whether ‘free’ or ‘guided’) should be taken into consideration when it comes to designing the follow-up experimental protocols for offline testing of the reported phenomena.

The second limitation of the real-time feedback approach comes from the variability of neural signals. Even if great care is taken to avoid changes in the subject's environment, there is still a high degree of variability in the neural responses even to identical external stimuli [63]. Interestingly, recent evidence suggests that neural variability should not be considered as ‘random noise’, but rather as intrinsic activity largely constrained by the functional organization of the brain [64]–[66]. This variability is indeed expected from a highly coupled, large-scale, non-linear system such as the brain [67]which responds differently to each novel external event depending on its ongoing state. For this reason, all the studies reporting task-related gamma band modulations have had to rely on averaging procedures across multiple repetitions of the same situation. This observed variability is lower in early sensory areas or in the motor cortex. For instance, the activity that can be recorded in the primary auditory cortex in human with intracranial EEG electrodes faithfully reflects the envelope of the auditory stimulus, with little noise [68]. However, the inter-trial variability is stronger in more associative brain regions, which may limit the interpretability of the ongoing signal by the patient in those areas. In addition, some of the spontaneous neural fluctuations are due to variations in certain cognitive processes, such as attentional and emotional fluctuations or random thoughts. Even though these processes often remain uncontrolled from an experimental point of view, it has been shown that the subjects can become aware of them [69]. It might therefore be possible for patients to learn to a certain extent to control and stabilize this component of neural variability. However, this is a hypothesis that has yet to be tested. We expect that the ability of the patient to correlate such fine elements of his/her own experience with his/her imaged brain activity would increase with the amount of time spent using our system. For this reason, in a future implementation, our system will be tested in a constant monitoring framework whereby the visualized time-varying power indices will be accessible to the implanted patient at all times as an additional TV channel. At will, the patient may decide to view the online power modulations switching through the different electrodes via the remote control. This so-called *Brain TV* set up would give the patients more time to become familiar with the system and yield observations of interest both for their presurgical evaluation and possible functional brain mapping hypotheses that can be later investigated in the presence of the experimenter and medical staff.

### Possible application to BCI research

Beyond its application to self-exploration, the feedback-based real-time power visualization is of direct interest to BCI research. First of all, the visual feedback of the spectral power of the ongoing cerebral activity computed in various frequency bands can be used to investigate the oscillatory parameters that achieve reliable/robust discrimination between different behavioral or cognitive states (a process known as feature extraction). Secondly, the real-time feedback can be used to train subjects to control certain parameters of their brain activity, such as power in a given band measured at a given area. This has been shown to be a fundamental aspect of most invasive and non-invasive BCI devices, with training periods being much shorter in the context of invasive recordings [38]. The proposed feedback of power modulations can of course be extended to the identification of novel parameters that might achieve a more reliable classification of mental state and/or intention.

Note that most BCI approaches also use low frequency components below 40 Hz. In this study, a strong correlation between neural power and behavior was reported in the high gamma band (60–140 Hz) supporting the idea of a possible relationship between an increase in gamma oscillations and task-specific cortical activations. But what about the other slower frequency ranges? The prominent role of local gamma band modulations which was apparent in our online power estimation sessions (and confirmed in the offline analysis) is in line with a growing body of evidence that indicates the existence of correlations between gamma-band power and the BOLD signal detected with MRI [70]–[72]. Nevertheless, it would be incorrect to assume that lower frequency components (such as theta, alpha and beta bands) do not contribute to the neural substrate underlying behavior. This has indeed been shown in various MEG and EEG studies and, most importantly, multi-band interaction between lower and higher frequencies also seems to play a major functional role [73], [74]. The real-time system introduced here readily allows for the simultaneous analysis of power emissions in different frequency-bands and may therefore also allow for the observation of task-related multi-frequency co-variations in real-time. Other parameters may also be monitored: we are extending the online estimation from power (an estimate of local synchrony) to long-range synchronization, using measures such as phase locking [75] in order to follow in real-time task-related variations the coupling between pairs of depth electrodes.

On the whole, the inherently exploratory nature of the investigation framework introduced by this paper differs from the standard experimental procedures that rely on an a priori defined hypothesis to design a paradigm that addresses a specific cognitive process. By contrast, beyond its possible clinical applications, the online investigation of power modulations (whether based on free or guided exploration) can be used to generate novel hypotheses which are particularly well examined with the implantation at hand. Furthermore, during the search for correlations between behavior and online power plots of a particular location various sensory, motor and cognitive functions can be tested. This opens up the possibility of detecting phenomena that could have remained otherwise unsuspected. Such exploratory approaches have been successfully applied in a number of animal studies yielding outstanding findings such as the discovery of mirror neurons in monkey [76], [77]. In general, the real-time experimental procedure proposed here leads to novel experimental paradigms that are adapted to the given implantation and suggest novel questions about the functional selectivity of certain brain areas that might have remained unknown otherwise. Moreover, the real-time experimental identification of a given phenomena based on real-time power imaging in single patients can lead to the design of systematic investigations with non-invasive imaging techniques (e.g. EEG, MEG) allowing for a more exhaustive spatial sampling of the task-related activations and the detection of large-scale functional networks.

In summary, the proposed procedure for real-time intracranial oscillatory power investigation and the results obtained with the first two patients lays out a blueprint for a novel approach to online dynamic brain mapping which, in conjunction with ECS and classical experimental paradigms, will hopefully improve the efficiency of the functional investigation during the relatively short presurgical evaluation period ultimately improving the post-operative outcome as well as our understanding of the dynamics of brain function.
